# Achieving spatially precise diagnosis and therapy in the mammalian gut using synthetic microbial gene circuits

**DOI:** 10.3389/fbioe.2022.959441

**Published:** 2022-09-02

**Authors:** Clare M. Robinson, Nina E. Short, David T. Riglar

**Affiliations:** Section of Structural and Synthetic Biology, Department of Infectious Disease, Imperial College London, London, United Kingdom

**Keywords:** synthetic biology, bacteria, gut, microbiota, diagnostic, therapy, spatial, biogeography

## Abstract

The mammalian gut and its microbiome form a temporally dynamic and spatially heterogeneous environment. The inaccessibility of the gut and the spatially restricted nature of many gut diseases translate into difficulties in diagnosis and therapy for which novel tools are needed. Engineered bacterial whole-cell biosensors and therapeutics have shown early promise at addressing these challenges. Natural and engineered sensing systems can be repurposed in synthetic genetic circuits to detect spatially specific biomarkers during health and disease. Heat, light, and magnetic signals can also activate gene circuit function with externally directed spatial precision. The resulting engineered bacteria can report on conditions *in situ* within the complex gut environment or produce biotherapeutics that specifically target host or microbiome activity. Here, we review the current approaches to engineering spatial precision for *in vivo* bacterial diagnostics and therapeutics using synthetic circuits, and the challenges and opportunities this technology presents.

## Introduction

The gastrointestinal tract is a spatially heterogeneous environment, with variable oxygen, pH, nutrients, host immune and antimicrobial factors, and microbiota composition (Donaldson et al., 2016; Tropini et al., 2017; Martinez-Guryn et al., 2019; Kudelka et al., 2020; Miller et al., 2021). Disease-associated changes to host and microbiome are also spatially heterogeneous, with variation seen both longitudinally and radially in the gut, for instance those detected during colorectal cancer (Abed et al., 2016; Saffarian et al., 2019) and Crohn’s disease (Gevers et al., 2014). These variations complicate disease understanding, diagnosis, and treatment. However, they can also be seen as an opportunity to develop location-specific diagnostics and therapies (Hua, 2020; Li et al., 2020).

The role of the gut microbiota in therapy is increasingly apparent: microbes modify and sequester orally delivered drugs, impacting efficacy on a patient-to-patient basis (Javdan et al., 2020; Klunemann et al., 2021); many drugs have unintended antibiotic effects, which can cause secondary complications as a result of the changed microbiota (Maier et al., 2018); and drug side-effects and toxicity can limit usage. Targeted therapies could offer increased efficacy, while reducing overall dosage, toxicity, and off-target effects. Disease diagnostics that provide spatial information could improve specificity and give the potential for differential diagnosis, thus reducing the need for invasive, costly procedures such as endoscopies. The development of spatially precise tools is hindered, however, by the gut’s inherent inaccessibility. Diagnostic sampling methods are either invasive (ex. endoscopies) or lack spatial resolution (ex. stool samples). Limited tools exist for spatially targeting therapeutic delivery.

A promising strategy to achieve these goals is using the bacteria residing in the gut for clinical applications. Bacteria have been used as probiotics to improve human health for over a century (Schultz, 2008) and, more recently, faecal microbiota transplants have also shown clinical success (Khoruts et al., 2021), but both lack definition and functional precision. Synthetic biology enables the design of engineered bacteria with specific diagnostic sensors and therapeutic outputs, with several examples having already entered clinical trials (Brennan, 2022).

**FIGURE 1:**
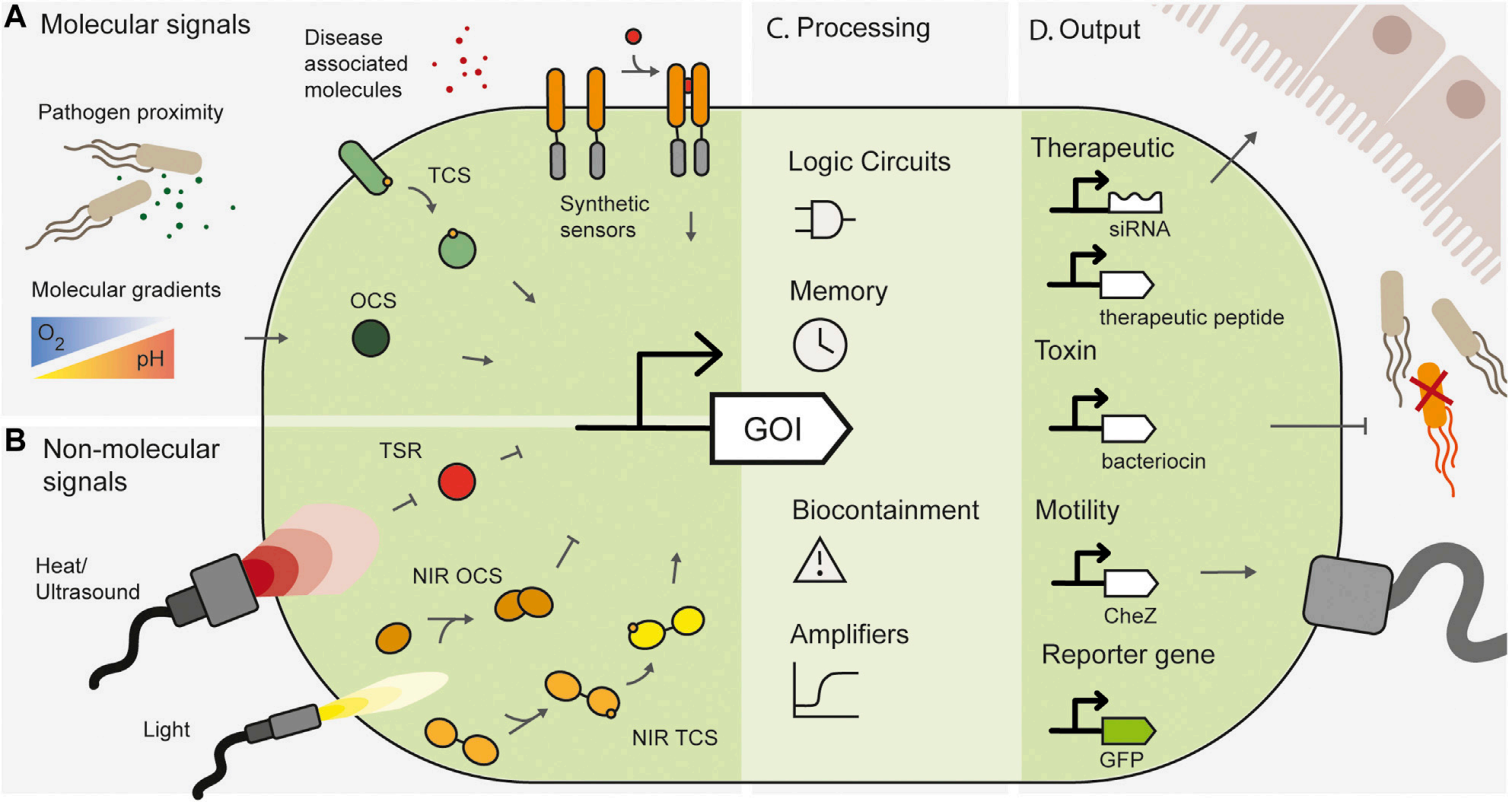
Overview of spatially specific engineered whole cell biosensors. **(A)** Spatially specific molecular signals such as proximity to pathogens, disease-associated small molecules or natural molecular gradients in the gut can be sensed by one-component systems (OCS), two-component systems (TCS) or synthetic engineered sensors. **(B)** Non-molecular signals such as heat and light can be sensed by temperature sensitive repressors (TSR), or light inducible systems such as near infrared sensitive one component systems (NIR OCS) or two-component systems (NIR TCS). **(C)** Synthetic gene circuits can add additional processing layers and functionality to engineered bacteria. **(D)** Engineered bacteria can produce useful outputs, such as therapeutic modules, pathogen targeting toxins, motility genes for spatial localization or reporter genes for biosensing.

This field is poised for rapid growth, with an expanding toolbox of synthetic biology tools available to engineer an ever-increasing range of bacteria (Ronda et al., 2019; Jin et al., 2022; Rubin et al., 2022). Basic synthetic circuits, some of which are detailed below, have been used to create *in situ* biosensors for diagnosis, recording, and therapeutic delivery (Figure 1).

• Binary memory systems can record the presence or absence of a stimulus using bistable transcriptional genetic switches (Gardner et al., 2000; Kotula et al., 2014), recombinases and/or integrases (Moon et al., 2011; Bonnet et al., 2012; Yang et al., 2014).
• Read and Write Memory Systems record signals by DNA editing, with “analogue” accumulation of edits in a population reflecting the presence, and combined strength, and/or duration of one or more stimuli. Examples include CRISPR base editing (CAMERA and DHARMA) (Tang and Liu, 2018; Tu and Esvelt, 2022 [pre-print]) and retron-based systems (SCRIBE and HiSCRIBE) (Farzadfard and Lu, 2014; Farzadfard et al., 2021).
• Stimulus Counters “count” small numbers of independent induction events using recombinases, riboregulated transcriptional cascades, or genetic transcriptional switches (Friedland et al., 2009; Stirling et al., 2020).
• Biocontainment Systems prevent engineered bacteria from surviving outside a specified environment. Examples include engineered physical containment (Tang et al., 2021), auxotrophy (Steidler et al., 2003), “kill-switches” (Stirling et al., 2017; Stirling et al., 2020; Rottinghaus et al., 2022) or xenobiotic approaches, in which genomically recoded organisms are dependent on synthetic molecules (Marlière et al., 2011; Mandell et al., 2015; Rovner et al., 2015).
• Logic Gates and Complex Signal Processing can be used to compute multiple signals, for example using recombinases or toehold switches (Green et al., 2014), enabling engineered complex logic, such as state machines (Ham et al., 2008; Roquet et al., 2016). They have been applied in the invertebrate gut (Gao and Sun, 2021).

For further detail we direct readers to recent reviews giving an overview of engineered bacterial biotherapeutics (Riglar and Silver, 2018; Cubillos-Ruiz et al., 2021; Brennan, 2022).

**FIGURE 2:**
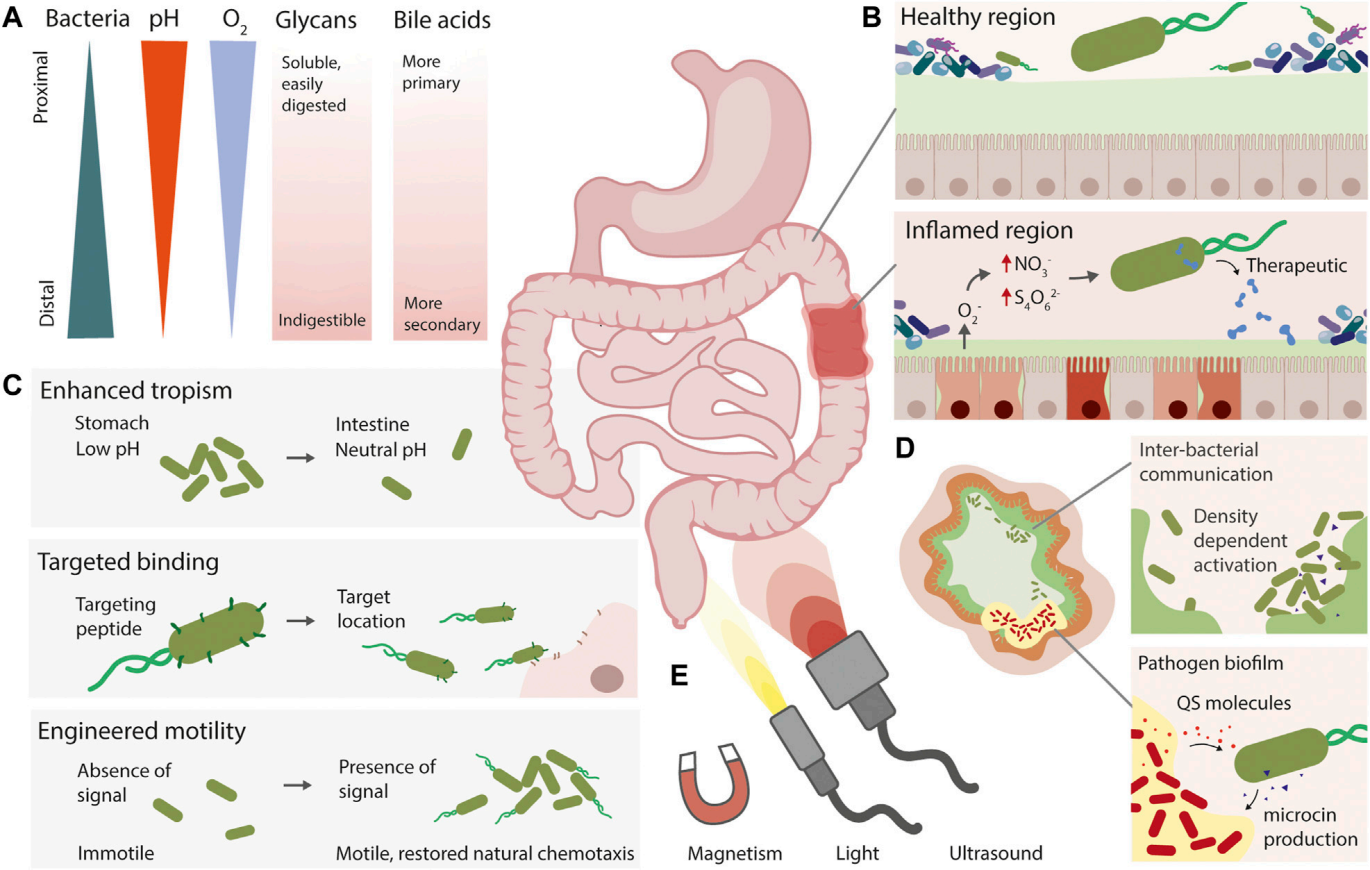
Strategies for engineering spatial precision in live diagnostic and therapeutic bacteria. **(A)** The gut is a heterogeneous environment, with natural gradients of different molecules and compounds. **(B)** Live bacteria can be engineered with the ability to detect signals associated with disease, such as nitrate and tetrathionate during inflammation, and produce a therapeutic output. **(C)** Synthetic approaches for spatially targeting engineered live bacteria include engineered or enhanced tropism, expression of targeting peptides, and engineering motility pathways. **(D)** Using quorum sensing to engineer inter-bacterial communication, or for close-range pathogen detection and eradication are complementary approaches to gain spatial resolution. **(E)** Non-invasive externally applied inducers could also allow precise spatial activation or detection of live therapeutics.

Herein, we focus on synthetic gene circuits that enable spatial precision within the mammalian gut (Figure 2). These circuits can augment the natural characteristics of a “chassis” strain, adding sensors for spatial cues, enhancing tropism and colonisation, or conferring the ability to naturally move towards specific regions of the gut. We will discuss spatially specific molecular “input” signals such as disease, pathogen, or endogenous gradients, externally controlled physical cues such as light, heat, or magnetic signals, and the opportunities and challenges in engineering spatially precise diagnostic and therapeutic bacteria.

## Spatial precision by molecular sensing

Bacteria have a naturally diverse catalogue of transcriptional, translational, or post-translational sensing mechanisms. These include, one-component systems (OCS), two-component systems (TCS), and extracytoplasmic function (ECF) sigma factors. Spatially precise and disease-specific gene regulators are used naturally by bacteria to control their function in the gut, making them an exciting option to control spatially precise functions of engineered bacteria. Synthetic signalling systems are also being developed to further expand this toolbox (Schmidl et al., 2019).

### Sensing physical proximity to pathogens or disease

Biosensing of infection and disease is a key goal for diagnostics but can also be used to activate synthetic circuits specifically at disease sites to create localised therapeutics. Many drugs, in particular broad-spectrum antibiotics, have off-target effects on the native microbiota, which can compromise colonisation resistance and promote opportunistic pathogen growth among other negative consequences (Maier et al., 2018; Javdan et al., 2020; Klunemann et al., 2021). The development of targeted therapies, whether by spatially restricting exposure or by other means, aims to mitigate these impacts.

Bacteria use quorum sensing (QS) to detect cell-to-cell proximity and density (Whiteley et al., 2017) over different spatial scales (van Gestel et al., 2021). QS is often used to design density- and proximity-dependent response due to its well-understood molecular mechanisms (Boo et al., 2021). In the context of the gut, spatial activation at infection sites can be achieved by repurposing pathogens’ own QS mechanisms (Saeidi et al., 2011; Gupta et al., 2013; Hwang et al., 2014; Hwang et al., 2017; Jayaraman et al., 2017). For example, *Escherichia coli* was engineered to detect N-acyl homoserine lactone (AHL) from the pathogen *Pseudomonas aeruginosa* and respond by lysing to release a toxin and a biofilm destabilising enzyme (Hwang et al., 2017). This system reduced *P. aeruginosa* load in a murine infection model by 98% when used prophylactically and 77% when administered therapeutically after infection (Hwang et al., 2017). While the therapeutic efficacy of this system was below that of traditional antibiotics, the strong prophylactic performance promises that further improvements, such as using more potent bacteriocins (Mills et al., 2017) or some of the measures below, could further increase efficacy. A version of the engineered strain lacking spatial activation failed to reduce faecal pathogen levels, demonstrating the importance of spatial specificity and the potential to reduce off-target effects using “sense-and-respond” approaches.

QS can also be used for density-dependent delivery mechanism, which are being actively developed in the context of tumours (Ganai et al., 2009; Din et al., 2016), and could assist engineered bacteria to spatially co-ordinate within the gut (Wu and Luo, 2021). Kim et al. (2018) demonstrated specific intra- and inter-species communication in the mouse gut using a QS-based circuit. Using QS for bacterial coordination may allow better control of function in the spatially partitioned gut environment (Whitaker et al., 2017; Wu et al., 2022).

DNA-responsive biosensors could offer a more specific signal of proximity to pathogens and other sites of disease. This may be particularly useful in situations where QS circuits suffer from crosstalk between strains. Two recent pre-prints have co-opted the natural competence of some bacterial strains to develop DNA-responsive biosensors (Cooper et al., 2021; Cheng et al., 2022). Cheng et al. (2022) developed a *Bacillus subtilis*-based system that specifically detects multiplexed environmental DNA sequences, including human pathogens within a community. An alternative system using *Acinetobacter baylyi* detected tumour-associated host DNA mutations within the mouse gut (Cooper et al., 2021). It remains to be seen whether these new biosensor mechanisms can deliver the sensitivity and stability necessary for more extensive use in the mammalian gut.

Live diagnostics have also been engineered to sense and respond to molecular disease biomarkers such as nitric oxide, nitrite and nitrate, thiosulfate, tetrathionate, and haeme (Archer et al., 2012; Courbet et al., 2015; Daeffler et al., 2017; Riglar et al., 2017; McKay et al., 2018; Mimee et al., 2018; Naydich et al., 2019). While the documented spatial resolution of these sensors is limited, these and other disease sensors could enable spatially and temporally precise “sense-and-respond” activation in a range of disease conditions. The localised activation and delivery of biological therapies in this way could minimise effective dosages, reducing the side-effects of otherwise toxic drugs.

### Sensing natural molecular gradients

Bacterial gene expression changes based on molecular cues that exist in natural gradients within the gut (Crook et al., 2020; Schmidt et al., 2022). These cues are often used by pathogens to spatially regulate function. Attaching and effacing pathogenic *E. coli* strains detect regional gut niches through hormones, oxygen, biotin, acetate, and bicarbonate to avoid activating metabolically burdensome virulence processes in unfavourable locations (Woodward et al., 2019). Clostridia modulate gene expression in response to spatially variable gut bile acids (Shalon et al., 2022 [pre-print]), and other microbes respond to specific spatially variable glycans (Ringot-Destrez et al., 2017). Similar sensing mechanisms could be co-opted to reduce the metabolic burden of therapeutic circuit expression in off-target regions. For example, a sensor for location within mucus layers would activate only in proximity to host cells and remain inactivated in the gut lumen. Spatial activation could also provide locational information in combination with disease diagnosis and monitoring.

Whilst promoters have been identified that exhibit consistent expression in *E. coli* throughout the mouse gut (Armetta et al., 2021), the catalogue of promoters with known spatial precision is limited. Chien et al. (2022) recently demonstrated spatial precision in the context of engineered growth tropism. Two *E. coli* strains were engineered to preferentially grow in specific regions of the mouse gut based on either hypoxic or low pH (Chien et al., 2022). The first showed enrichment in the hypoxic large intestine, whereas the second was depleted. Although promising, the resolution of this approach is currently limited to large gut regions. Understanding spatial characteristics of a greater range of promoters is therefore important for increasing precision and allowing control in all regions of the gut. Promoters responding to alternative molecules that vary in the gut, such as bile acids, short chain fatty acids, glycans or immune signals or used in concert with higher-order signal processing and logic gates may be particularly promising avenues to achieve this goal.

### Engineering tissue tropism, targeted binding, and pseudotaxis

Localised activity can also be achieved by spatially restricting bacterial growth, which is possible through natural tropisms of a chosen chassis strain, engineered tropism through expression of targeted binding proteins, and through non-genetic mechanisms such as the adherence of antibodies to the bacteria’s surface (Vargason et al., 2020) or using encapsulation methods to contain (Liu et al., 2018; Mimee et al., 2018; Tang et al., 2021; Inda et al., 2022 [pre-print]) and selectively release bacteria within different regions of the gut (Cui et al., 2021). Some bacteria express outer-membrane proteins that bind to specific molecules or subsets of mammalian cells, concentrating them in the locations in which these cells or molecules are enriched. For example, *Helicobacter* species bind specific glycans in the small intestine and stomach epithelium (Matos et al., 2021), and *Fusobacterium nucleatum* bind a host factor overexpressed in colonic adenomas (Abed et al., 2016), both causing enrichment. These binding strategies can be repurposed in engineered biotherapeutics. Examples of targeted binding include a binding protein from *Streptococcus gallolyticus* expressed in *E. coli* to target cancerous colorectal epithelial cells (Ho et al., 2018), synthetic adhesins expressed in *E. coli* allowing specific adherence and colonisation of solid tumours (Piñero-Lambea et al., 2015), or recombinant curli fiber fused trefoil factors which enhanced binding to mucins and mammalian cells in cell culture (Duraj-Thatte et al., 2018) and in the mouse gut (Praveschotinunt et al., 2019).

Targeted binding can also simultaneously activate a desired gene of interest. Chang et al. (2018) created bacterial sensors to novel ligands by using single-domain antibodies fused to DNA-binding domains, and used this system to detect bile salts in the serum of liver transplants patients (Chang et al., 2021). Targeting proteins designed to be expressed on the surface of bacteria must be able to be effectively secreted or displayed, which may pose engineering challenges (Salema et al., 2013).

Another method for spatial targeting involves placing key motility genes under the control of a sensor module to direct the biotherapeutic to a target site. Placing the *E. coli* chemotactic protein CheZ under the control of hydrogen peroxide, nitric oxide, or quorum molecule sensitive promoters in a CheZ KO strain allowed “pseudotaxis” towards the respective signals (Hwang et al., 2014; McKay et al., 2018; Virgile et al., 2018). Although an intriguing idea, the effectiveness of motility-based targeting remains to be demonstrated in the mammalian gut, where forces such as peristalsis may impact bacterial localisation.

## Spatial precision by sensing non-molecular signals

A powerful complementary approach to control engineered bacteria is using externally applied physical stimuli, such as light, temperature, or electromagnetic fields. As with the molecular sensing approaches described above, these tools aim to create minimally disruptive, non-invasive methods to activate diagnostics and therapeutics within specific regions of the gut. Unlike the molecular approaches, physical stimuli could be directed externally by a health care professional or an individual for on-demand activity.

### Optogenetics

Optogenetic systems use light-sensitive proteins or protein domains to regulate downstream cellular processes upon exposure to specific wavelengths of light (Lindner and Diepold, 2021). They are reversible, minimally invasive, and do not require overly specialised equipment, making them potentially well suited for clinical applications. Bacterial optogenetic systems have been tested in invertebrates (Hartsough et al., 2020) and smaller mammalian models, but not in deep tissue contexts in larger mammals. Systems that respond to near-infrared II (NIR-II, >1,000 nm) light are best suited for deep tissue contexts, such as spatial targeting within the gut, owing to deeper penetration, reduced scattering and lower phototoxicity of NIR-II light compared to shorter wavelengths, and their capacity for spectral multiplexing (Shcherbakova et al., 2018; Chen et al., 2020; Qiao et al., 2021). Several engineered bacterial TCS that respond to red or NIR light exist (Levskaya et al., 2005; Tabor et al., 2009; Tabor et al., 2011; Ma et al., 2017; Ong et al., 2018; Ong and Tabor, 2018; Hartsough et al., 2020). A recent OCS based on a NIR-sensitive phytochrome enabled transcriptional repression in *E. coli* with a 115-fold dynamic range (Kaberniuk et al., 2021), and benefits from being smaller than TCS counterparts.

Technological advances in photonics for medical applications are accelerating the use of optogenetics towards clinical contexts. For example, the poor tissue penetration of visible/UV light can be overcome using upconversion nanoparticles, which convert an externally applied NIR stimulus into internal UV or visible light (Chen et al., 2020). This approach enabled NIR-light inducible expression of anti-inflammatory molecules in murine ulcerative colitis models (Yang et al., 2020; Cui et al., 2021). Implanted light-sources have also been used for optogenetic control of deep-brain and gut (Hibberd et al., 2018; Gong et al., 2020), and biodegradable hydrogel-based waveguides have been used to deliver light across >5 cm of mammalian tissue (Feng et al., 2020). To confirm the translational potential of bacterial optogenetic systems, testing in larger mammalian models is required, with careful consideration being given to the system’s light-sensitive proteins and their cofactors, spatial and temporal resolution, kinetics, dynamic range, phototoxicity and potential for multiplexing. A growing number of optogenetic tool databases will help facilitate this (Lindner and Diepold, 2021).

### Temperature

Temperature-sensitive systems can also provide non-invasive spatially precise circuit induction. Recently, focussed ultrasound was used to control release of immune checkpoint inhibitors for anti-cancer immunotherapy in a murine solid tumour model (Piraner et al., 2017; Abedi et al., 2022). The mechanism was based on ultrasound induced local tissue heating which activated a temperature-sensitive transcriptional repressor, TlpA (Piraner et al., 2017). Ultrasound has also been used to image bacterial enzyme activity in the mouse gut using bacteria expressing an acoustic biosensor (Lakshmanan et al., 2020). Furthermore, focussed ultrasound is already being successfully used to treat human patients in other contexts, such as malignant liver, prostate, kidney, and pancreatic cancer (Izadifar et al., 2020), demonstrating the possibilities of ultrasound and temperature-sensitive activation in spatially resolved gut diagnostic and therapeutic capacities.

### Magnetism

Magnetism is an alternative external physical inducer commonly used in the clinic in the form of MRI. Whilst magnetic-sensitive genetic circuits have been developed in eukaryotic cells (Mosabbir and Truong, 2018; Madderson et al., 2021), they have not to our knowledge been established in bacterial contexts. Nonetheless, certain bacteria are naturally magnetosensitive, containing magnetic crystals that enable them to sense and orient themselves in alignment to magnetic fields (Faivre and Schüler, 2008) and alter MRI contrast (Liu et al., 2016). Magnetism can therefore be used to direct magnetotactic bacteria towards target sites, such as for delivering drugs to a tumour environment (Felfoul et al., 2016), and may also offer opportunities for MRI-based spatial diagnostics.

## Discussion

### Limitations and opportunities

The catalogue of spatial sensors that have been tested and validated in the mammalian gut is limited. New sensors can be developed through genome mining, direct targeted design, or directed evolution of existing sensors to improve or change their desired specificity or sensitivity (Miller et al., 2022). Bacteria have multitudes of regulatory sensing mechanisms which have begun to be catalogued in databases such as RegPrecise (Novichkov et al., 2013), Prodoric (Dudek and Jahn, 2022), MISTdb (Gumerov et al., 2020), P2TF (Ortet et al., 2012) and CollecTF (Kilic et al., 2014). In addition, high quality bacterial transcriptome analysis databases, such as iModulonDB (Rychel et al., 2020), are available and can provide additional regulatory information for biosensor construction. However, we have yet to take advantage of the huge natural catalogue of sensors in part due to the limited toolbox available for understanding spatial variation within the gut. Difficulties in acquiring samples with spatial information (ex. by biopsy, endoscopy, or dissection rather than faecal sampling), a lack of healthy human donor samples, and challenges performing transcriptomics of the gut microbiota have all hindered our fundamental understanding of this important area of biology. Developing new tools and prioritising datasets to understand the microbiota’s functional biogeography in both animal models and humans is thus important. Promisingly, recent years have seen a steady increase in multi-omic and biopsy sampling being undertaken in human clinical studies, especially those including transcriptomics and metabolomics, which should provide new targets of interest for biosensor development and clinical validity. High throughput screening pipelines also have the potential to accelerate biosensor discovery (Sheth et al., 2017; Schmidt et al., 2018; Naydich et al., 2019; Schmidt et al., 2022).

An additional fundamental challenge is that any biosensor will be inherently limited by the combination of the spatial precision of the signal, the temporal characteristics of the synthetic circuits, and movement of bacteria within the gut. Small molecules that readily diffuse from their target site may activate distant sensors in a less targeted manner. Studying engineered probiotic activity *in situ* with high spatial precision techniques when possible, or integrated engineered memory systems when not (Riglar et al., 2017; Naydich et al., 2019; Schmidt et al., 2022), will help to assess these factors. Alternatively, the exogenous, non-molecular signals discussed above may circumvent some of the issues associated with endogenous signals. Similarly, tapping into mechanisms like chemotaxis that bacteria natively use to deal with noisy molecular gradients may be a useful approach in comparison to more binary “present-on/absent-off” approaches. These characteristics will ultimately depend on a set of complex factors including the molecules in question, the application of interest and the local microbiota, which can deplete signalling molecules differentially. Differences in gut anatomy, diet, motility and retention time which vary between vertebrate species, individuals, disease states, and even time of day will also play a role given their known impacts on the microbiota. Ultimately, the precision required for a given system will depend on the application, such as the degree of off-target toxicity of the therapeutic in question. The fact that pathogens have managed to solve these issues through evolution to tightly control virulence gene expression within humans despite this level of noise, however, is heartening.

Engineered biotherapeutics are a promising technological opportunity for spatially precise investigations of the gut, and for diagnosis and treatment of gut diseases. While the field is still in its infancy, particularly with respect to clinical delivery, spatially precise engineered microbes are already primed to serve as exciting scientific investigative tools for non-invasive sampling of the complex gut microenvironment in animal models (Riglar et al., 2017; Naydich et al., 2019; Schmidt et al., 2022). There are undoubtably additional barriers for the use of these tools over traditional approaches in the clinic. However, we expect that the unique advantages of spatially precise engineered biotherapeutics, ranging from *in situ* non-invasive diagnosis, to complex signal processing power, and targeting for reduced off-target effects, will offer worthy rewards for overcoming these in a range of clinical scenarios. Advances in spatial and functional precision will need to go hand-in-hand with advances in biosafety, biocontainment and patient acceptance that are already being tackled more broadly for the use of engineered bacteria as live biotherapeutic products (Brennan, 2022). The next decade is likely to include critical milestones in this space, and regulatory approval of the first products for clinical use will provide frameworks for addressing these challenges (Cordaillat-Simmons et al., 2020). Technological advances in synthetic biology, decreasing DNA synthesis cost, and an increasing understanding of the gut and gut microbiota will continue driving discovery in this area and development of new effective diagnostics and treatments for disease.
